# Think Out of the Box, Think Out of the Eye Reappraisal of HIV/AIDS Retinopathy

**Published:** 2014

**Authors:** Francesco Pichi, Paolo Romani, Paola Carrai, Paolo Nucci

**Affiliations:** 1 San Giuseppe Hospital – University Eye Clinic, Milan, Italy; 2 Azienda Sanitaria Locale di Bologna, Bologna, Italy

**Keywords:** human immunodeficiency virus (HIV), retinopathy, Highly Active AntiRetroviral Therapy (HAART)

## THE PAST --OUT OF THE BOX

In 1981, acquired immunodeficiency was proposed denominator of a newly defined syndrome of diseases that were on the rise in promiscuous male homosexuals and intravenous drug users (1). The reference to acquired immunodeficiency syndrome (AIDS) as a new disease (2,3), instead of a new syndrome composed of known diseases, inspired a search for a single new pathogen (2). In 1983 Montaigner and coworkers (4) suggested that pathogen to be lymphadenopathy-associated virus [now termed human immunodeficiency virus(HIV)] and Gallo et al. (5) believed human T-cell leukemia virus (HTLV) infection to be the cause of AIDS.

In April 1984 the US Secretary of Health and Human Services announced that HIV was the cause of AIDS, and an antibody test for HIV, termed the “AIDS test”, was registered as a patent by Gallo and collaborators (6,7). This happened even before any American study on HIV had been published (6), and has given rise to an HIV/AIDS denialism movement since. David Rasnick, an US AIDS nonconformist, and his colleagues University of California biologist Peter Duesberg and Charles Gechekter, were the loudest proponents of the theory that HIV could not be the cause of AIDS (8,9), and that AIDS was not an infectious disease at all. On the contrary, they postulated that recreational drugs, such as nitrate inhalants, antivirals such as zidovudine (AZT), and immune hyperstimulation were the exact causes (8). They were supported by Kary Mullis, who was Nobel prize laureate for chemistry for the discovery of the polymerase chain reaction (PCR) technique, sharing his own skepticism based on the inability to find a single widely-accepted scientific citation for the statement that HIV is the probable cause of AIDS. Moreover, there was not any clear evidence supporting sexual transitiveness of AIDS. “Modern science is based on the ability to demonstrate facts to the audience, no matter how skeptical those facts sound” he said.

In particular, Duesberg argued that HIV did not fulfill Koch’s postulates for identifying the causes of infectious diseases. In 1884 Robert Koch, discoverer of the anthrax bacillus, presented a group of conditions, termed Koch’s postulates for identifying the causes of infectious diseases. We present them herein in a very concise form, with Dusberg’s (counter) arguments:

1) The agent occurs in each case of disease and in the amount sufficient to cause pathological effects.

According to Duesberg, HIV violated this first postulate since free virus could not be documented in T lymphocytes of the peripheral blood of most people with AIDS. However, the HIV--T-cell specificity has lost ground since then, as HIV can often be detectable only in monocytes (10). Another tenet holding the case against HIV was that avirus that could be found in only 1 in 400 peripheral blood lymphocytes could not possibly engender immunodeficiency. This, of course, contradicts the fact that blood, while being the easiest tissue to sample, is not the only one infected by HIV, so that we know nowadays that during the latent phase virus is filtered and trapped by dendritic cells of peripheral lymphoid tissue. Nowadays, the epidemiological concordance of HIV exposure and AIDS has been clearly shown in a number of different studies that document the presence of HIV or HIV antibodies in over 95% of AIDS patients (11–21). Moreover, Duesberg declared that AIDS could not have an infectious etiology because “viruses and bacteria work fast or never”. Here, he was mixing up viral replication at the cellular level with disease in the infected individuals. By his logic, treponema does not cause tertiary syphilis; hepatitis B virus does not cause chronic liver disease, and so on.

2) The agent can be isolated and propagated outside the host.

In the 1980s, HIV isolation, although possible in up to 80% of AIDS cases, was technically very difficult. Nowadays, multiple HIV isolates are cultured, and HIV has been cultivated in fresh human T lymphocytes and macrophages. It is true that virus isolation is sometimes not successful, but the presence of HIV can be demonstrated by PCR amplification even of low abundance HIV genomes in most AIDS patients. Duesberg moreover believed that viruses do not cause disease after specific neutralizing antibodies appear in the blood. Therefore, HIV could not cause AIDS. In fact, the persistence of antibody’s titers for over a decade in AIDS patients is most likely explained by the continued stimulation of the humoral (antibody producing) immune system by low levels of sequestered virus. If not, antibody titer would drop off, as it does for other diseases when the pathogen is cleared.

3) The transfer of the agent to an uninfected host leads to disease.

Duesberg denied Koch’s third postulate stating that all attempts to cause AIDS in chimpanzees had been unsuccessful, even after they have been antibody-positive for 4 to 5 years. The postulate of transmission pathogenesis cannot be fulfilled by epidemiological data, but instead is a requirement for direct empirical evidence. Ethical reasons preclude experimental transmission to uninfected human patients (except of Duesberg, who said to be considering the to imitate Max von Pettenkofer, who drank a *Vibrio cholerae* culture), making verification difficult. Therefore, this has been the most controversial Koch’s postulate with respect to HIV and AIDS.

Epidemiology has been proposed as adequate to identify causative agents, particularly in human diseases where Koch’s postulates are difficult to meet, as in the case of HIV. The evidence that HIV causes AIDS is epidemiological and virological, not molecular. In every country and city where AIDS has appeared, HIV infection preceded it just by a few years; in every social group at risk of AIDS, HIV has preceded the disease.

## THE PRESENT -- OUT OF THE EYE

*AIDS/HIV Retinopathy*

Even with the eye being among the most common sites of involvement (22–27), this HIV/AIDS denialism movement surprisingly bypassed the ophthalmic community.

Retinopathy is common and is generally classified as either noninfectious (AIDS-associated micro- and macrovasculopathy; HIV-associated neuroretinal disorder) or infectious (28–30).

Microvasculopathy is described as the most common ocular manifestation of AIDS (31). Clinical findings may be recognized in the conjunctiva, retina, and optic disk. Retinal microvasculopathy is clinically known for cotton-wool spots located in the posterior pole. Micro-aneurysms are clinically not often obvious, but occasionally may be visible on the fluorescein angiogram. Cotton-wool spots have rounded borders, are not associated with significant amounts of adjacent hemorrhage (although scattered dot and blot hemorrhages may be present), and do not enlarge. The optic disk may develop pallor consequent to microvascular occlusion. Most microvasculopathy patients are asymptomatic (32). Histopathologically, findings of AIDS microvasculopathy bear a resemblance to those of diabetic retinopathy. Retinal vascular abnormalities include necrosis of pericytes, endothelial cell swelling, and thickened basement membranes. The pathophysiology of the vascular injury is controversial. Hypotheses include immunoglobulin deposition, endothelial cell infection by HIV, and hyperviscosity.

Retinal macrovasculopathy, accompanied by large vessel occlusions, including noninfectious branch retinal artery occlusions and central and branch retinal vein occlusions (31) are extremely unusual.

HIV-associated neuroretinal disorder is characterized by subtle vision abnormalities –visual field loss, reduced contrast sensitivity, altered color vision, and diminished visual acuity—all in the absence of ocular opportunistic infections (33–34). The proportion of patients with visual functional abnormalities among HIV-positive individuals appears to be higher than that of the general population, even in the absence of any apparent ocular pathology or severe immunosuppression. Belonging to social sub-cultural minorities, history of intravenous drug use, lower Karnofsky score, anemia, and lack of private health insurance, all bring about a significantly increased risk of visual field loss. Kalyani et al have described a structural basis for this disease, correlating worsening contrast sensitivity and color vision with temporal thinning of the peripapillary retinal nerve fiber layer (RNFL) using spectral domain optical coherence tomography (SD-OCT) in HIV-positive patients without history of ocular disease.The RNFL and the unmyelinated prelaminar optic nerve have high concentrations of mitochondria, required for the energy intensive maintenance of axonal membrane potentials and axonal transport. There is evidence suggesting that HIV infection itself or its treatment (specifically, treatment with certain nucleoside reverse transcriptase inhibitors) is toxic to mitochondrial function, resulting in skeletal myopathies, peripheral neuropathies, insulin resistance, and life-threatening lactic acidosis. They suggest that the smaller caliber axons comprising the maculopapillary bundle are more susceptible to mitochondrial toxicity and resultant axonal loss, mediated by antiretroviral therapy, leading to the visual functional decline associated with neuroretinal disorder.

Posterior segment opportunistic infections develop either in a pattern of a necrotizing retinitis or a unifocal or multifocal choroiditis. Retinitis is more prevalent than choroiditis. Except toxoplasmosis, cryptococcus, and syphilis, which may present as either retinitis or choroiditis, opportunistic agents are included in a differential diagnosis specific to the clinical presentation of the infection. Necrotizing retinitis may be clinically further subdivided based upon the extent of associated ocular inflammation. Retinitis in inflamed eyes usually occurs in patients with higher CD4+ counts and is more commonly due to acute retinal necrosis, toxoplasmosis, syphilis, or late stages of Cryptococcus spp. infection. Retinitis in quiet eyes occurs in patients with lower CD4+ counts and is more commonly due to cytomegalovirus (CMV) and progressive outer retinal necrosis.

*Highly Active AntiRetroviral Therapy (HAART)*

When two or more nucleoside reverse transcriptase inhibitors are combined with either a protease or a non-nucleoside reverse transcriptase inhibitors, HIV replication is suppressed more efficiently than with any drug class alone. The combination regimen is termed highly active antiretroviral therapy (HAART) and its potency is related to the lower likelihood of the development of viral resistance to antiretrovirals (35, 36). HIV protease is a crucial proteolytic enzyme involved in the cleavage of viral polyproteins and production of functional protein products during the late stages of HIV replication. Protease inhibitors lay off HIV replication by blocking the cleavage of the Gag-Pol polypeptide through occupation of the active site of HIV protease, leaving so immature virus, incapable of infecting new CD4 cells (1). In 1996 protease inhibitors were approved for treatment of patients with HIV and currently include saquinavir (Fortovase), saquinavir mesylate (Invirase), ritonavir (Norvir), amprenavir (Agenerase), lopinavir (Kaletra), indinavir sulfate (Crixivan), nelfinavir mesylate (Viracept), and atazanavir sulfate (Reyataz). Before protease inhibitors were introduced, the mainstays of HIV therapy were the nucleoside and non-nucleoside reverse transcriptase inhibitors, both of which suppress HIV replication by inhibiting the RNA directed DNA polymerase, reverse transcriptase (37). Nucleoside reverse transcriptase inhibitors are nucleoside analogs and include zidovudine (Retrovir), stavudine (Zerit), lamivudine (Epivir), abacavir (Ziagen), didanosine (Videx), zalcitabine (Hivid), and emtricitabine (Emtriva). Non-nucleoside reverse transcriptase inhibitors include efavirenz (Sustiva), nevirapine (Viramune), and delavirdine mesylate (Rescriptor). In patients who respond to HAART, a rise in CD4+ counts and sustained suppression of HIV replication may occur (38). Recovery of the immune system with HAART results from increased absolute CD4+ cell counts, first by an increase in memory T cells and later by renewed production of naïve CD4+ T cells (39). If suppression of HIV replication lasts for long enough, lymphoproliferative responses may be restored even in patients with previous severe immunodeficiency (40), restoring natural protection against many opportunistic infections (41).

*Is HIV the cause of AIDS manifestations in the eye?*

If Duesberg were an ophthalmologist, he would argue that manifestations of HIV infection in the eye are different from AIDS manifestations.

Logically, posterior segment opportunistic infections are strictly correlated with the AIDS-associated lymphopenia rather than HIV infection itself. In 1996, the introduction of HAART rapidly and dramatically shifted the trajectory of the HIV/AIDS epidemic, increasing life expectancy and decreasing the incidence of opportunistic infections and malignancies. (42, 43). Prior to the HAART era, the lifetime incidence of CMV retinitis in HIV/AIDS patients approached 30%; the rate of new cases has dropped 80–90% with HAART(44–49). The importance of HAART and its resultant effect on CD4+ count in controlling progression or reactivation of CMV retinitis cannot be overemphasized; in fact, in patients whose immune system is reconstituted (CD4+ count ≥ 100 cells/mL at two or more consecutive visits at least 6 months apart), HAART is probably more important than targeted anti-CMV treatment.

However, other ocular opportunistic infections, such as ocular syphilis, appear to be unaffected (50). HAART has a less dramatic effect on the epidemiological and clinical aspects of the ocular syphilis in HIV-infected patients compared to that observed with CMV retinitis. In many respects, ocular syphilis continues to be an undiscriminating disease, irrespective of CD4+ count and immunodeficiency (51–55). Furthermore, HAART has no effect at all on the incidence of HIV micro- and macrovasculopathies, which, Duesberg would say, are a consequence of HIV infection of the retinal endothelial cells and pericytes rather than a part of the AIDS spectrum. Duesberg’s hypothesis that AIDS may be caused by intravenous drug use and antiretroviral therapy seems to have at least a part of truth when it comes to the eye, since the retina is among the most metabolically active tissues, making it a prime target for unwanted side effects of chemotherapeutic agents. Since the introduction of HAART, for example, a new pathological entity termed HIV-associated neuroretinal disorder has been detected in the eye. These subtle vision abnormalities have been associated with minority status and history of intravenous drug use, but at the same time it has been demonstrated that antiretroviral therapy, which is toxic to mitochondrial function, damages the RNFL and the unmyelinated prelaminar optic nerve which have high concentrations of mitochondria.

## CONCLUSIONS

Nowadays there is clearly no doubt that HIV, if not adequately controlled with antiretroviral therapy, is the cause of a severe lymphopenia which opens the door to a wide spectrum of opportunistic infections and tumors that constitute as a whole the spectrum of AIDS. However, Duesberg would surely have been very fascinated by the posterior segment manifestations of HIV infection in the eye. Some of these are caused directly by HIV damage to retinal vessels (and therefore should be referred as HIV-vasculopathy, instead of AIDS-vasculopathy). Some are sequelae of HAART toxicity (a point to Duesberg).Finally, others have nothing to do with direct HIV infection but are caused by opportunistic infections which gain access to the eye when the T lymphocytes count drops (AIDS-related infectious retinitis).

## DISCLOSURE

Conflicts of Interest: None declared.
